# Successful Combination of Ulvans and Thermal Spring Waters from as Burgas (Ourense) for Hydrating Cosmetic Creams in Healthy Volunteers

**DOI:** 10.3390/md24080275

**Published:** 2026-08-06

**Authors:** Isa B. D. Ingrez, María Milagros Fernández-Varela, María Dolores Torres, Natalia Fariñas-Valiña, José Gantes, Manuela Buján, Antonio Muiños, Herminia Domínguez, María Reyes Pérez-Fernández

**Affiliations:** 1 Porto-Muiños. S.L., Polígono Industrial O Acevedo, Parcela 14, Cerceda, 15185 A Coruña, Spain; laboratorio1@portomuinos.com (I.B.D.I.); imasd1@portomuinos.com (M.B.); antonio@portomuinos.com (A.M.); 2 CINBIO, Department of Chemical Engineering, University of Vigo, Campus Ourense, 32004 Ourense, Spain; matorres@uvigo.gal; 3 Escuela Universitaria de Enfemería, SERGAS, Ramón Puga 52-54, 32005 Ourense, Spain; maria.milagros.fernandez.varela@sergas.es (M.M.F.-V.); nfarval@sergas.es (N.F.-V.); mperfer1@sergas.es (M.R.P.-F.); 4 Campus Auga, University of Vigo, Rúa Canella da Costa da Vela, 12, 32004 Ourense, Spain; 5 Cosmeticos Anfer S.L., c/Parroquia de Bergondo, Parcela R-10, Shed 2, Bergondo Industrial Park, Bergondo, 15165 A Coruña, Spain; laboratorio@anfercosmeticos.com

**Keywords:** creams, rheology, color, functional and biometric, ulvan

## Abstract

Different hydrating cosmetic creams were formulated with ulvans obtained by a previously optimized process, consisting of hydrothermal treatment and a solvent-free concentration stage to obtain ulvans with 80% purity in carbohydrates, 34% rhamnose, 11% sulfate and >10 kDa. The incorporation of low-mineralization thermal spring waters in the formulation of hydrating creams with 1% ulvans and green ingredients caused a shear thinning behavior that was more marked than in creams formulated with distilled water. As Burgas thermal spring water caused the largest decay, suggesting an improvement in the cream’s spreadability on the skin. This spring water was selected for formulating and bottling a commercial hydrating cream that was further evaluated regarding both physicochemical and biometric properties with a group of 120 healthy volunteers to assess the performance of both ulvans and As Burgas thermal spring water. The results of both objective and subjective evaluation indicate the enhanced skin hydration provided by ulvan and thermal spring water, as well as their favorable additive effect. A high tolerance was observed because all tested creams decreased the tightness sensation, and scaling was significantly reduced in the ulvan group. The findings confirm the potential additive skin benefits of combining ulvan with thermal spring water in hand cream formulations.

## 1. Introduction

Polysaccharides constitute the major fraction in marine algae and can be incorporated as functional and bioactive ingredients in cosmetics, complying with the increasing demand for natural and sustainable products [1]. Carbohydrates can be utilized in cosmetics as moisturizing and thickening agents and also as promising bioactives for their various properties beneficial to skin, including antioxidant, anti-melanogenic, and skin anti-aging properties [2].

*Ulva* spp. are worldwide cultivated seaweeds, with established food uses, and can also be available from green tides or algal blooms, which are increasingly frequent events [3]. Ulvans are the predominant polysaccharide in green seaweeds presenting a unique composition with sulfated L-rhamnose, uronic acids (D-glucuronic, L-iduronic), and xylose [4]. Ulvans are gaining increasing commercial interest for medical and cosmetic applications based on their biological properties [5] and mechanical features, showing potential as functional biopolymers that could be useful in drug delivery, regenerative medicine, novel materials, and tissue engineering [6]. The conformation of ulvans in a solution is pH dependent and can be influenced by the presence of counter-ions, and the gel forming properties of ulvan are greatly affected by the pH and ions in the medium [7].

Different studies have confirmed the potential of ulvans for skin care based on the antioxidant, anti-inflammatory, anti-aging, hydrating, and regenerative properties determined by their composition and structure. Whereas both poly- and oligosaccharide fractions from *Ulva* spp. obtained by enzyme-assisted extraction and depolymerization enhanced cell proliferation on human dermal fibroblast [8], crude ulvans (57 kDa) reduced fibroblast proliferation rate, and the depolymerized fraction (4 kDa) had no significant effect [9]. Furthermore, incorporation at 1–5% yielded creams with adequate rheological and optical properties [10].

Mineromedicinal spring waters have different benefits on the skin due to their unique chemical composition, and may exhibit the ability to repair and strengthen the skin barrier and improve antioxidant and immune defenses, which counteract aggressions on the skin [11,12]. Thermal spring waters may have a role in the treatment of dermatological diseases and for certain skin conditions with immunomodulatory and inflammatory components [13,14,15]. The mechanisms involved include chemical, thermal, mechanical, and immunological effects, and the chemical composition of thermal water is crucial. Improved skin hydration has also been reported following the application of mineral-rich water, such as Vichy volcanic mineralizing water, which has been shown to accelerate epidermal cell turnover and improve sensitive skin hydration, with soothing and decongestant effects, reducing dryness and erythema [11], and also for low-mineral-content water, which protects the stratum corneum from dehydration and maintains skin surface ultrastructure without inducing mechanical stress [12]. The contribution of the hydrobiome seems to be determinant, and thermal spring waters and algae extracts are some of the most promising ingredients for cosmetic purposes, especially for the treatment of reactive skin and other skin alterations [16].

Mineromedicinal spring waters can be valuable bioactive ingredients in cosmetic creams [15], the effect of different ions can be also determinant in the rheological properties of the product. The creams formulated with Monfortinho thermal water in order to improve skin hydration and relieve psoriasis symptoms exhibited apparent viscosity values significantly different from those of creams prepared in distilled water [17]. Similarly, in mixtures of hyaluronic acid from the Yumoto Hot Spring, Nakajima et al. [18] observed enhanced hydrating capacity due to the improved diffusion of this active element, as well as the formation of new bonds in a more flexible structure. Therefore, it is expected that the incorporation of thermal spring water in cosmetic products formulated with ulvans may confer interesting physicochemical properties, but no information regarding their combination is found in the literature. Complying with the need for innovative, efficient, and green extraction techniques [1], a hydrothermal based process has been developed [19] and a solvent free ultrafiltration stage was applied to recover both intermediate and high-molecular-weight fractions [20]. In the present study, the hydrothermally obtained ulvans that were further refined to concentrate the >10 kDa fraction were used for the formulation of cosmetic creams that were tested for stability and hydrating potential, objectively assessed by biometric properties and by subjective feeling in healthy volunteers.

## 2. Results and Discussion

### 2.1. Characterization of the Concentrated Product

#### 2.1.1. Chemical Composition of Concentrated Ulvan

The characterization of ulvan is shown in Table 1. The final product contains 10.9% moisture and exhibits pH 6.03, slightly acidic, which should be compatible with skin pH. As expected, rhamnose is the major constituent (33.88% dry basis). The presence of glucose suggests that the slightly acidic conditions during the extraction could have solubilized other components, since, in addition to ulvan, *Ulva* spp. also contains cellulose, xyloglucan, and starch [21]. The biochemical characterization confirmed that the ulvan product used has a carbohydrate purity of 80% and is predominantly composed of a repeated ulvanobiouronic acid disaccharide [20]. The refining technology does not allow for selective protein separation from oligosaccharides, and the final product contained 7.48 g of protein/100 g ulvan product. Sulphate content was 10.6% and ash almost 22%. These ranges compare favorably with other commercial ulvans tested for different topical and therapeutic uses. Comparable ash content and sulfate content was reported for depolymerized ulvan [9], but higher ash (30%) [22] and higher sulfate (19.6–48.3%) and uronics (17.6) and lower carbohydrate content with rhamnose (26.2) have also been reported [22,23,24]. However, the reported protein content was lower (1–4%), particularly after protease treatment [9,23]. The anti-aging skin properties of *Ulva pertusa* aqueously extracted polysaccharides was favored by the highest sulphated group and uronic acid content, but hydrothermal operation provides lower contents than ultrasound assistance [25]. The pH in the rejected permeate was slightly higher, 6.82 ± 0.47 g/100 g, but the lipidic content was comparable (0.92 ± 0.13 g/100 g). Ash, sulphate, and protein content were higher than in the retentate product, 59.65 ± 0.75, 34.14 ± 3.43, and 8.37 ± 0.45 g/100 g, respectively. The ethanol precipitable fraction was also lower: 25.29 ± 6.89 g/100 g.

#### 2.1.2. Structural Characteristics

**Figure 1:**
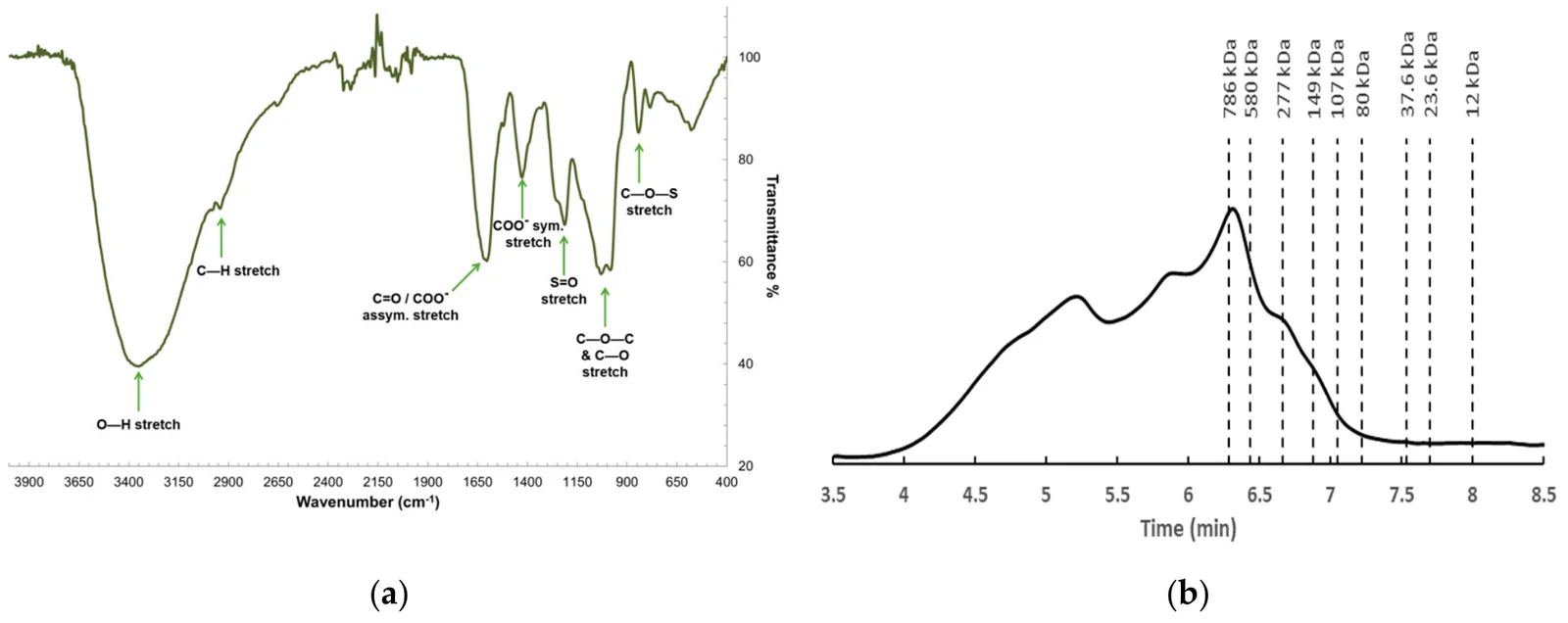
(**a**) FT-IR spectrum and (**b**) HPSEC of the ulvan-concentrated product using polyethylene oxide standards (molecular weight between 23.6 and 786 kDa) (Agilent, Santa Clara, CA, USA).

The FT-IR analysis of the purified ulvan extract Figure 1a revealed the characteristic absorption bands of sulphated polysaccharides typical of *Ulva* spp. The broad and intense band at 3280–3400 cm^−1^ corresponds to O–H stretching vibrations of hydroxyl groups widely distributed along the polysaccharidic backbone. The weaker peak near 2942–2983 cm^−1^ was assigned to aliphatic C–H stretching of –CH_2_ and –CH_3_ groups, confirming the carbohydrate nature of the material. A clear absorption band at 1604–1612 cm^−1^, together with a shoulder around 1427 cm^−1^, is attributed to the asymmetric and symmetric stretching vibration of carboxylate groups (–COO^−^) from uronic acids, confirming the presence of ulvanobiuronic acids. The relative intensities of these two peaks suggest moderate uronic acid content. The absorption at 1210–1230 cm^−1^ corresponds to the S=O asymmetrical stretching of the sulphated esters, indicative of the presence of sulphated rhamnoses in ulvan. Complementary bands observed at 843 cm^−1^ and 785 cm^−1^ correspond to the C–O–S stretching of sulphate groups linked to the C-3 position of rhamnose units (axial and equatorial vibrations, respectively). The simultaneous presence of these three sulphate-related bands indicates that the sulphation pattern of ulvan was preserved during extraction and purification procedures. Nevertheless, the overall spectra shows evidence of the coexistence of uronic acid and sulphate functional groups, suggesting a moderate degree of sulphation. The broad complex region between 1030 and 985 cm^−1^ is assigned to C–O–C and C–O stretching vibrations of glycosidic linkages, representing the main polysaccharidic backbone of rhamnose, xylose, and glucuronic acid units. These bands are typically among the most intense absorptions in ulvan spectra and serve as reference points for semi-quantitative comparison of functional groups. At lower wavenumbers (<700 cm^−1^), weak peaks at 578–604 cm^−1^ are likely be related to skeletal vibrations of sugar rings. Altogether, the FT-IR profile confirms that the >10 kDa concentrate fraction retains the structural signature of ulvan, with well-defined hydroxyl, carboxylate, and sulphate functional groups, suggesting potential conjugation with proteins and low content in lipid residues. These findings align with previous ulvan spectral characterizations in the literature [20,26,27,28,29,30].

A broad distribution of molecular weight was observed in the chromatogram of Figure 1b, with a major proportion at 700 kDa. Ulvan with a MW distribution centered at 1000 kDa was extracted from *Ulva rigida* with comparable technology and proposed for nanofibrous patches for wound healing treatment [24]. Lower molecular weights could provide different skin-related effects, i.e., crude (57 kDa) ulvans showed different metabolic activities on human dermal fibroblasts than depolymerized (4 kDa) ulvans [9].

### 2.2. Influence of Thermal Spring Water on Rheology

**Figure 2:**
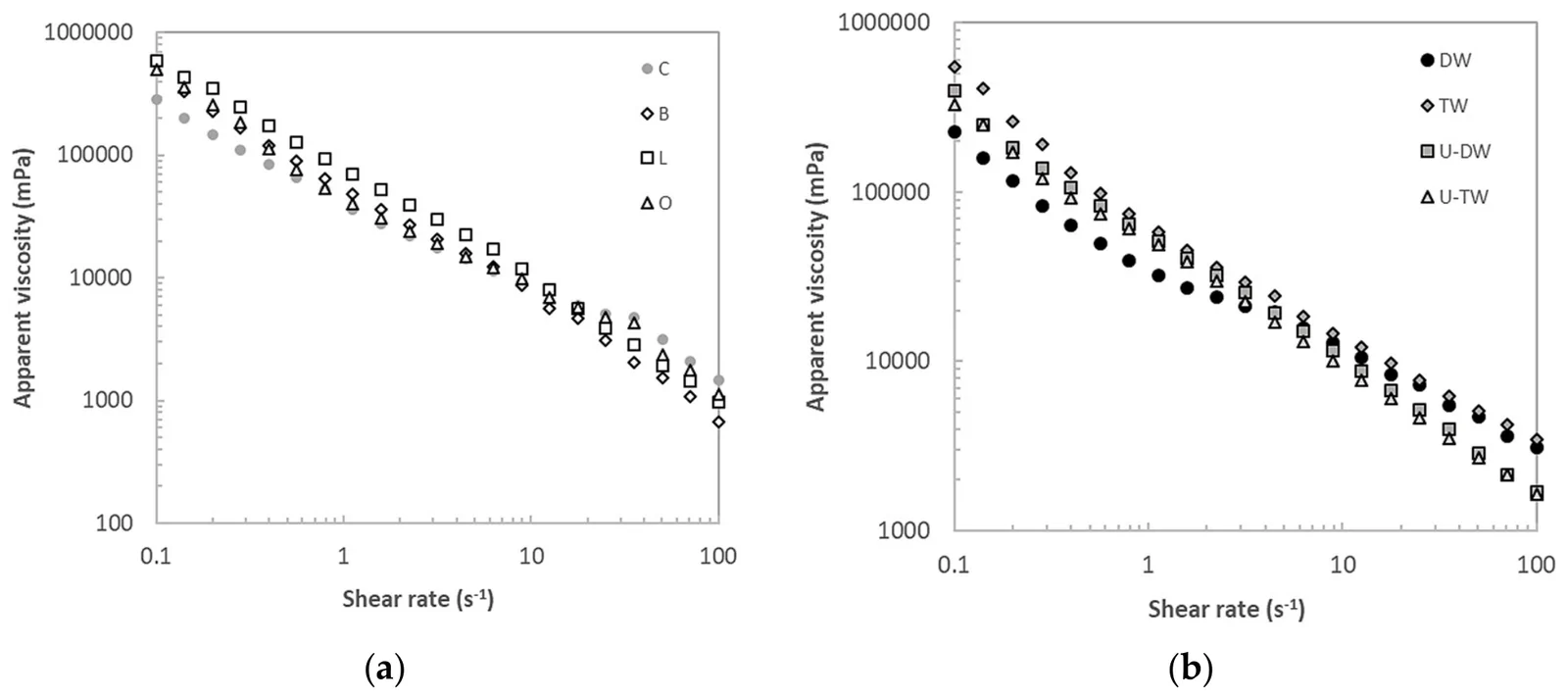
Apparent viscosity at 25 °C of (**a**) the preliminary set of the creams with As Burgas (B), Laias (L) and Outariz (O) thermal spring waters; (**b**) creams developed in distilled water (DW), As Burgas thermal spring water (TW), ulvan-based creams incorporated with distilled water (U-DW), and ulvan-based creams incorporated with As Burgas thermal spring water (U-TW).

Figure 2a shows the effect of the selected waters on the apparent viscosity of the preliminary set of formulated creams with green ingredients. All samples exhibited shear-thinning behavior, as evidenced by a pronounced decrease in apparent viscosity of approximately three orders of magnitude with increasing shear rate. The largest decay was observed for those creams prepared using As Burgas thermal spring water. Analyzing the obtained profiles, those creams formulated with this water suggested enhanced characteristics from the applicability (related to the highest shear rates) point of view, favoring their spreadability on the skin [31]. Also, the highest stability in terms of apparent viscosity was identified for these samples with a negligible variation in viscosity over the 6-month study period. It should be highlighted that neither hysteresis nor water syneresis was identified for the studied period. These are two relevant advantages from the processing, storage and application point of view. In all cases, the magnitude of the apparent viscosity is consistent with those previously reported for similar creams [32]. The pH was stable for at least 6 months, with values of 6.5 for those formulated with DW, 7.6 for those formulated with As Burgas, 7.2 for those with Laias water, and 7.4 for those with Outariz waters; in the latter case, there was 1.5% variation.

Figure 2b displays the formulated creams prepared with As Burgas thermal spring water in the presence of ulvan. Again, all samples presented similar shear-thinning behavior, displaying profiles consistent with their commercial counterparts [31]. It should be highlighted that creams incorporated with ulvan or thermal water exhibited higher apparent viscosities at the largest shear rates when compared with the control cream made with distilled water. Conversely, at shear rates above 5 s^−1^, the opposite trend was observed in ulvan-containing creams, irrespective of the type of water used. Although the low-shear viscosity qualitatively followed the sulfate concentration of the thermal waters, this trend was not maintained at high shear rates; therefore, the rheological differences cannot be conclusively attributed to sulfate or to any individual mineral. Consequently, those creams enriched with thermal waters hinted at improved characteristics from the processing (linked to low shear rates) and applicability (link to high shear rates) [31]. Following this response, the ulvan-based creams also could enhance the spreadability on the skin and, consequently, consumer acceptance. In this case, creams enriched with both thermal water and ulvan exhibited viscosity stability when storage at room temperature for one year of study. It should be pointed out that these creams offered the advantage of lack of hysteresis phenomenon and water syneresis in the tested period.

### 2.3. Characterization and Stability of the Commercial Bottled Hydrating Creams

The ulvan was dried to allow for storage and further mixture with different thermal spring waters, but in an alternative approach, the retentate stream could be incorporated in the creams in a ready-to-use approach to avoid drying cost [33]. The creams are white and shiny, and the oil-in-water (O/W) emulsion, with water as the external phase, ensures rapid initial absorption and a cooling feeling.

The incorporation of ulvan at 1% resulted in slightly lower, although non-significant, lightness value in the creams. Yellowness also was reduced with the addition of ulvan and by the use of thermal spring water. Regarding the rheological properties, ulvan increased the apparent viscosity of creams formulated with distilled water, but decreased it in those prepared with thermal spring water (Table 2). However, Guidara et al. [10] used ulvan concentration from 1 to 3% (*w*/*w*) of enzymatic–chemical extract and acidic extract and found enhanced lightness, stability, thixotropic and viscoelastic behaviors. At higher concentrations, they found enhanced storage stability, strength, and firmness, together with increased lightness and more pronounced pseudoplastic behavior. No microbial growth was detected following repeated application.

### 2.4. Clinical Study with Healthy Volunteers

**Figure 3:**
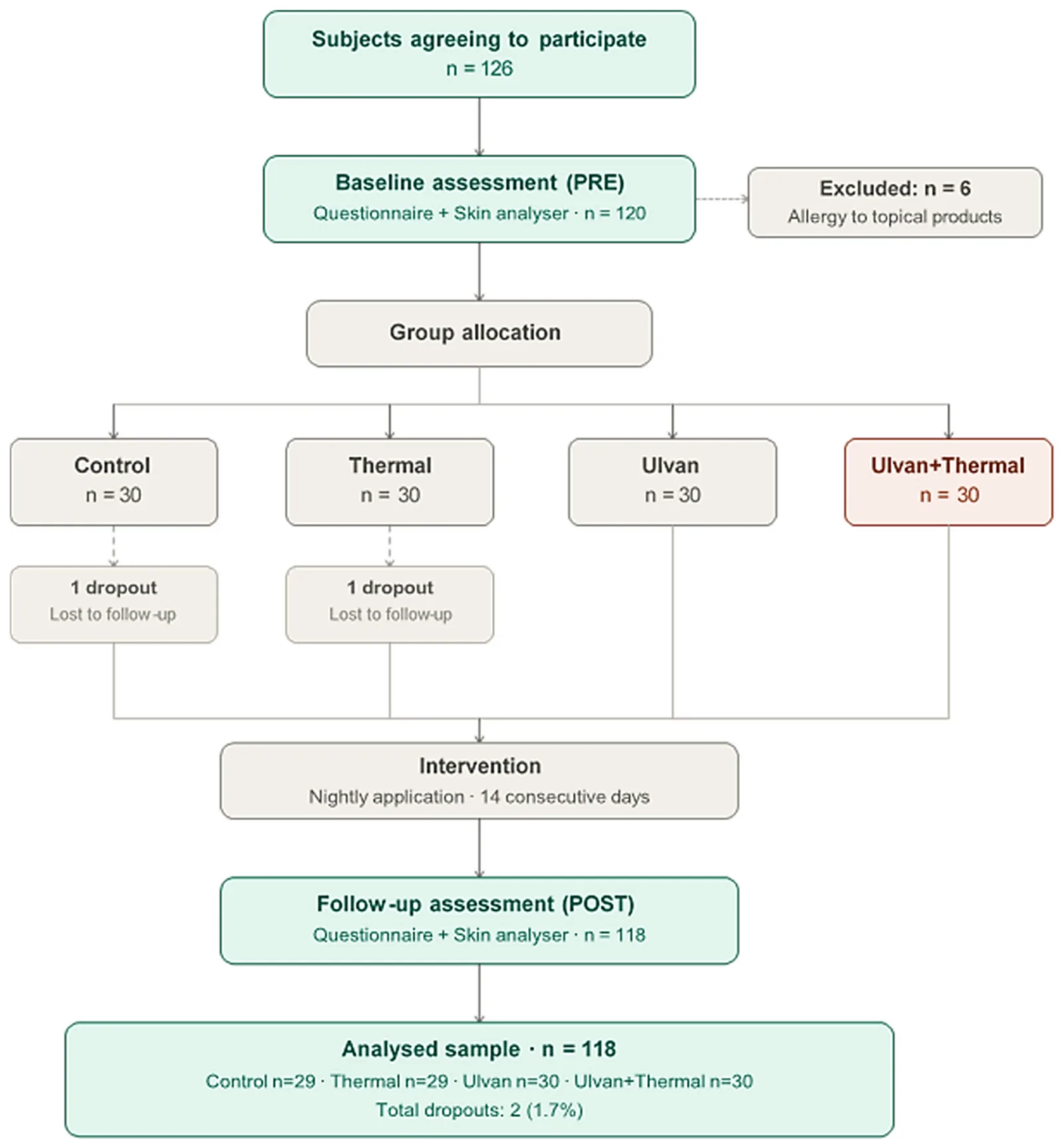
Flow diagram of the quasi-experimental, pre-post pilot study conducted with healthy volunteers (*n* = 126 enrolled; *n* = 6 excluded due to allergies to topical products; *n* = 2 voluntary dropouts during intervention). Final analyzed sample: Control (*n* = 29), Thermal (*n* = 29), Ulvan (*n* = 30), and Ulvan+Thermal (*n* = 30). Note: The shaded group (Ulvan+Thermal) constitutes the primary experimental group of the study.

Of the 126 subjects who agreed to participate in the study, 6 were excluded due to allergies to topical products. The remaining 120 participants completed the initial assessment and were assigned to the four study groups (*n* = 30 per group). During the intervention, there were two voluntary withdrawals (1.7%), one in the Control group and one in the Thermal group, both due to failure to attend the final assessment. The analyzed sample consisted of 118 participants: Control (*n* = 29), Thermal (*n* = 29), Ulvan (*n* = 30), and Ulvan+Thermal (*n* = 30). The study flow diagram is presented in Figure 3.

All formulations were presented to participants in identical dispensers, differentiated only by an identification code known exclusively by the investigators, ensuring the complete blinding of participants with respect to the assigned product. Group allocation was performed consecutively in order of recruitment.

#### 2.4.1. Characteristics of the Volunteer Sample

The majority of participants were women (90.7%; *n* = 107), with a mean age of 26.5 ± 12.8 years (median: 21 years; range: 18–63), reflecting the predominantly student composition of the university community linked to the Nursing Degree program where the study was conducted (85.6% students, 5.1% faculty, and 9.3% administrative and service staff). In total, 65.3% of participants routinely used gloves, a particularly relevant finding given the healthcare context of the sample. Smoking was reported by 9.3% of participants (*n* = 11), distributed similarly across groups.

The four groups were homogeneous in all baseline variables analyzed: age (H = 0.157; *p* = 0.984), gender (χ^2^ = 1.059; *p* = 0.787), occupation (χ^2^ = 1.012; *p* = 0.985), frequency of handwashing (χ^2^ = 7.892; *p* = 0.545), regular use of hand cream (χ^2^ = 10.844; *p* = 0.093), and use of gloves (χ^2^ = 0.077; *p* = 0.994), confirming the homogeneity of the groups at the beginning of the study (Table 3).

#### 2.4.2. Objective Skin Hydration

At baseline using the Corneometer, all four groups showed similar hydration levels (H = 0.809; *p* = 0.847; η^2^ = 0.007), with medians between 38.0 and 41.0 AU, all within the dry category (30–45 AU) [34], confirming that the groups started from a comparable initial state (Table 4). After 14 days of intervention, only the experimental groups showed significant improvements in objective hydration. The Control group remained stable (median: 39.0 AU in PRE and POST; Z = −1.265; *p* = 0.206), while the Thermal (Z = −4.718; *p* < 0.001; r = 0.876), Ulvan (Z = −4.705; *p* < 0.001; r = 0.859), and Ulvan+Thermal (Z = −4.766; *p* < 0.001; r = 0.870) groups exhibited a significant improvement and a large effect size in all three cases. Analysis of the individual increase in hydration (∆ PRE→POST) revealed significant differences between groups (H = 90.248; *p* < 0.001; η^2^ = 0.771), with a large effect size, indicating that the assigned formulation is the main determinant of the observed improvement (Table 5). The Control group experienced no change (median: 0.0 AU; IQR: 0.0–0.0). The Thermal and Ulvan groups showed increases of 4.0 AU (IQR: 3.0–7.0) and 8.5 AU (IQR: 4.3–11.8), respectively. The Ulvan+Thermal group showed the greatest increase, with a median of 28.0 AU (IQR: 23.3–30.8), representing a shift from the dry category to the normal/hydrated category.

Post hoc analysis using Mann–Whitney U with Bonferroni correction (α = 0.0083) confirmed that the Ulvan+Thermal group showed a significantly higher increase in skin hydration when compared to the Control group (U = 28.0; Z = −6.342; *p* < 0.001; r = 0.826), the Thermal group (U = 29.0; Z = −6.169; *p* < 0.001; r = 0.803), and the Ulvan group (U = 45.0; Z = −5.993; *p* < 0.001; r = 0.774). The Thermal and Ulvan groups also outperformed the Control group (*p* < 0.001 in both cases). Nevertheless, no significant differences were identified between Thermal and Ulvan (U = 251.0; Z = −2.801; adjusted *p* = 0.030), suggesting that both active ingredients individually produce equivalent improvements.

Increased hydration after 24 days was also reported for wound dressings prepared with ulvan and marine gelatin on the burn-inflamed skin of SKH-1 hairless mice [23], and different studies used ulvan alone or in combinations with other materials in different wound dressing formulations [35]. However, studies with creams are less common [10].

#### 2.4.3. Comparison Between Objective Hydration and Subjective Perception

When comparing the instrumental measurement with self-perception using equivalent categories—very dry, dry, and normal/hydrated in both (Table 6)—it was observed that, at baseline, participants systematically overestimated their hydration level. This discrepancy was particularly marked in the Ulvan and Ulvan+Thermal groups, where 90.0% and 70.0% of the subjects perceived their skin as normal/hydrated at baseline, compared to their corresponding objective hydration of 26.7% and 13.3%, respectively.

At post-test, the objective improvement was significantly greater than the subjective one. In the Ulvan+Thermal group (POST), 96.7% of subjects achieved the normal/hydrated category in the objective measurement (compared to 3.3% at baseline—PRE), while in self-perception, the percentage increased from 70.0% to 96.7%. The Control group showed no relevant changes in either measurement.

These findings align with previous evidence attributing various biological functions to ulvan [36], including the improvement in parameters related to hydration and skin barrier function [37], as well as with studies indicating that the combination of specific thermal waters and algae extracts constitutes a promising strategy in the dermocosmetic field [16]. Furthermore, the Thermal and Ulvan groups showed no statistically significant differences between them at the post-intervention time point (U = 251.0; adjusted *p* = 0.030), indicating that neither of the two active ingredients alone produced the same level of hydration as their combination. The Ulvan+Thermal group was significantly superior to both the Thermal group (U = 29.0; *p* < 0.001; r = 0.803) and the Ulvan group (U = 45.0; *p* < 0.001; r = 0.774), with large effect sizes in both cases.

This pattern suggests an additive effect between ulvan and sodium bicarbonate mineral water, such that the combination of both active ingredients generates a response superior to what would be expected from either of them separately. To formally assess this, we performed the Scheirer–Ray–Hare test (the non-parametric equivalent of a two-way ANOVA), which confirmed significant main effects for both ulvan (H = 60.561; df = 1; *p* < 0.001) and thermal water (H = 33.787; df = 1; *p* < 0.001), but a non-significant interaction term (Ulvan × Thermal Water: H = 0.035; df = 1; *p* = 0.851), confirming that the observed effect is additive rather than synergistic in the strict statistical sense.

Regarding effect sizes, Rosenthal’s r values obtained for the intragroup Wilcoxon comparisons were large in all of the experimental groups (r ranging from 0.631 to 0.876). As noted by Field [38], the value of r depends on the study design, measurement error, and data variability, and is therefore not directly comparable across studies. In the context of this study, the low intragroup variability—particularly in the Control group, where virtually all participants showed no change (delta median: 0.0 AU; IQR: 0.0–0.0)—and the large between-condition differences contribute to high Z statistics and, consequently, large r values. This is consistent with a design in which participants were selected with objectively dry skin at baseline and in which the intervention produced a uniform directional response within each group [38].

The independent contribution of each active ingredient to the observed additive effect may be explained by distinct mechanisms. Ulvan, as a sulfated polysaccharide, forms hydrophilic films through hydroxyl groups, uronic acid carboxylates, sulfates, and ionic bridges, conferring high swelling capacity and water retention in the stratum corneum [39,40]. Furthermore, rhamnose-rich ulvan fractions are structurally similar to connective tissue glycosaminoglycans and may support fibroblast viability and extracellular matrix synthesis, contributing to long-term barrier quality [8]. Independently, thermal spring waters have been reported to improve key epidermal barrier markers such as filaggrin and defensin-related responses, with direct benefits on skin hydration [41]. The additive effect observed in the Ulvan+Thermal group is therefore consistent with two complementary but independent mechanisms acting simultaneously on the stratum corneum. These mechanisms remain hypothetical and require experimental confirmation through future studies, including TEWL measurement, skin barrier biomarker analysis, and penetration studies.

#### 2.4.4. Subjective Perception of Hydration

At the start of the study, the groups were comparable in their self-perceived hydration (H = 1.641; *p* = 0.650; η^2^ = 0.014). After the intervention, intragroup analysis showed that the Control group did not perceive any changes (Z = −0.533; *p* = 0.564), while the three experimental groups did notice a significant subjective improvement: Thermal (Z = −3.397; *p* < 0.001; r = 0.631), Ulvan (Z = −4.197; *p* < 0.001; r = 0.766), and Ulvan+Thermal (Z = −3.771; *p* < 0.001; r = 0.689).

The intergroup comparison in the POST revealed significant overall differences (H = 25,420; *p* < 0.001; η^2^ = 0.217). Post hoc analysis showed that all three experimental groups perceived significantly higher levels of hydration than the Control group: Thermal (U = 204.5; Z = −3.359; adjusted *p* = 0.002; r = 0.441), Ulvan (U = 171.0; Z = −4.003; *p* < 0.001; r = 0.521), and Ulvan+Thermal (U = 212.5; Z = −3.374; *p* < 0.001; r = 0.439). No significant differences were observed between the three experimental groups themselves (adjusted *p* ≥ 1.000 in all comparisons). Detailed subjective perception results are expressed in Table 7. However, subjective perception did not discriminate between the three experimental formulations (adjusted *p* ≥ 1.000). This dissociation between objective and subjective measurements is a well-known phenomenon in cosmetic research [42] and underscores the importance of combining instrumental and self-reported outcomes in efficacy studies.

#### 2.4.5. Skin Tolerance

Skin tolerability was assessed by evaluating tightness, scaling, and irritation before and after the intervention.

**Tightness:** All four groups started from a homogeneous baseline distribution (H = 1.233; *p* = 0.745). All groups showed a reduction in the sensation of tightness after the intervention (*p* between 0.005 and 0.013), with no significant differences between groups at the post-intervention time point (H = 1.748; *p* = 0.626). This pattern suggests that the reduction in tightness can be partly attributed to the non-specific effect of skin hydration produced by the topical application itself, independent of the active ingredient, which is consistent with the known relationship between stratum corneum hydration and skin comfort [43] (Table 8). However, the Ulvan+Thermal group presented the largest within-group effect size (r = 0.519), which could indicate a trend toward a greater reduction in tightness with the combined formulation.

**Scaling:** Baseline scaling frequencies were similar in all groups (Control: 27.6%; Thermal: 24.1%; Ulvan: 30.0%; Ulvan+Thermal: 23.3%). Only the Ulvan group showed a statistically significant reduction (exact McNemar test: *p* = 0.031), decreasing from 30.0% to 10.0% (Table 9). The Thermal and Ulvan+Thermal groups showed positive trends that did not reach statistical significance (*p* = 0.062 and *p* = 0.125, respectively), probably due to the low prevalence of scaling in the sample, which limits the test’s ability to detect statistically significant changes. The comparison between groups in the post-test showed no significant differences (χ^2^ = 5.737; *p* = 0.125; V = 0.220).

Irritation: Baseline irritation frequencies were low in all groups (between 13.3% and 20.7%). The Ulvan+Thermal group was the only one that achieved complete absence of irritation at the end of the intervention, with a reduction from 13.3% to 0.0%, although this did not reach statistical significance (exact McNemar test: *p* not calculable due to the absence of discordant pairs). The comparison between groups in the post-intervention phase showed no significant differences (χ^2^ = 2.037; *p* = 0.565; V = 0.131), confirming a favorable tolerability profile for all formulations (Table 9).

Given that moisturizers play a fundamental role as adjuncts in the management of common skin conditions—such as atopic dermatitis, contact dermatitis, psoriasis, acne, and rosacea [43]—it is important to consider selecting products that combine efficacy, safety, and suitability for each patient’s skin condition. In this context, the findings of our study provide preliminary information on natural formulations that could be integrated into daily skin care routines, especially in populations with frequent exposure to irritants such as repeated handwashing and the habitual use of gloves, which are characteristics that are present in the analyzed sample. Furthermore, to date, specific data on the impact of the waters from the As Burgas spring on these skin parameters are not available, so the results obtained open up a promising line of research in the field of Galician thermal cosmetics.

## 3. Materials and Methods

### 3.1. Seaweed and Thermal Spring Water

Biomass of *Ulva* spp. was harvested in September 2019 from the eastern Atlantic coastal area of Illa de Arousa (O Grove, Galicia, Spain) and subsequently transported to the facilities of Porto-Muiños (Cerceda, A Coruña, Spain) for industrial processing. Upon arrival at the laboratory, the biomass was washed and air-dried at temperatures below 38 °C to preserve thermolabile bioactive compounds, until a moisture content below 15% was achieved. The dried biomass was then chopped, milled, and converted into a fine powder. Samples were stored in sealed containers under ambient conditions until further use for ulvan extraction.

Thermal spring water from (B) As Burgas [UTM ETRS89 coordinates X = 593459, Y = 4687561 and UTM zone = 29], (L) Laias [UTM ETRS89 coordinates X = 579996, Y = 4686411 and UTM zone = 29] and (O) Outariz [UTM ETRS89 coordinates X = 589130, Y = 4688900 and UTM zone = 29] (Ourense, Spain) were collected in clean and sterilized glass bottles of 1 L, transported for 3 h at room temperature and stored at 4 °C before use. The mineral composition is shown in Table A1.

### 3.2. Experimental Methodology

#### 3.2.1. Extraction and Purification of Ulvan

**Figure 4:**
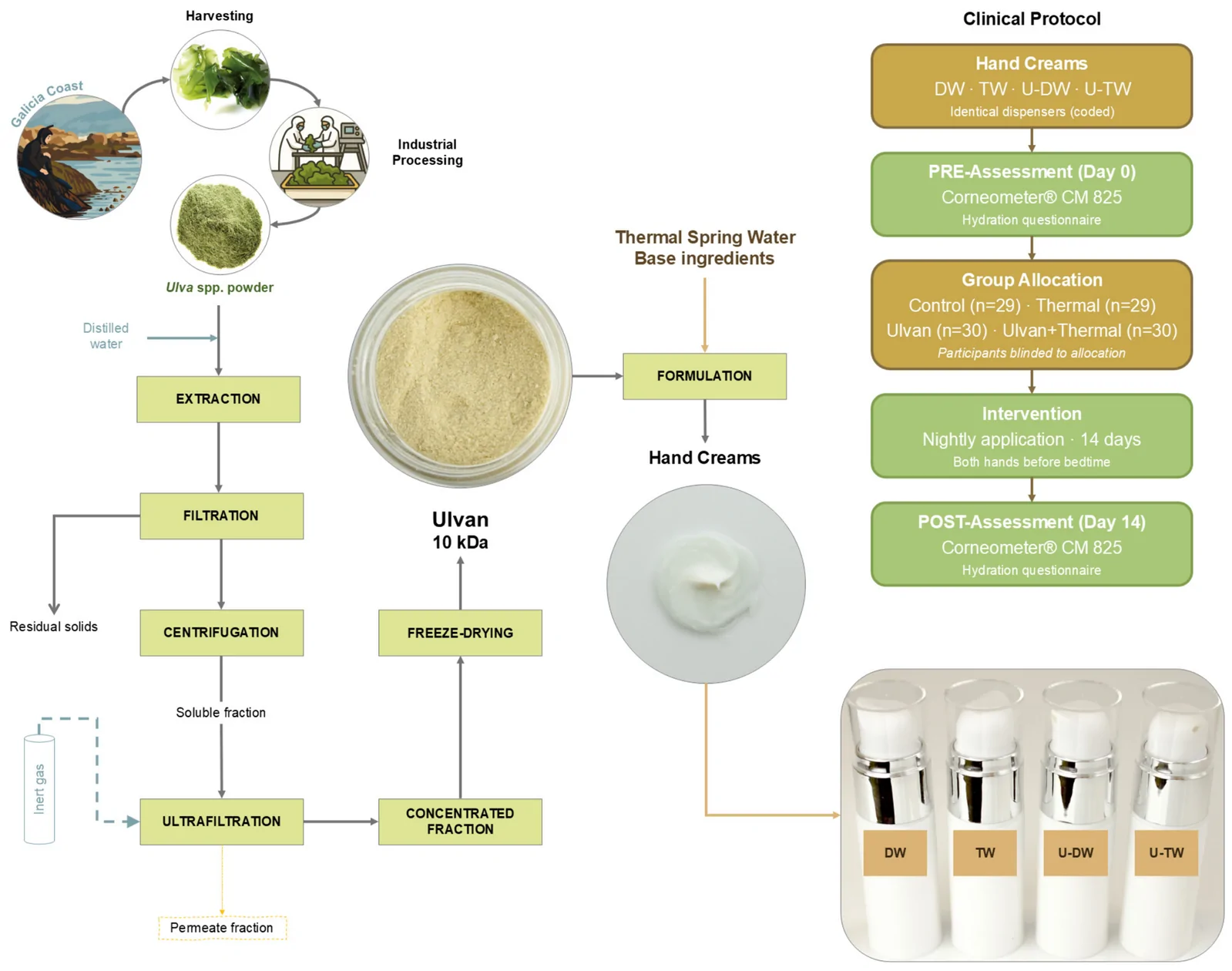
Schematic representation of the experimental workflow from *Ulva* spp. extract preparation to product development and clinical evaluation, including the 14-day nightly application protocol and PRE/POST assessment time points.

Concentrated ulvan extract was produced in accordance with food safety production guidelines. *Ulva* spp. biomass was suspended in distilled water at a ratio of 1:30 (*w*:*w*) and subjected to hydrothermal treatment at 121 °C for 135 min using a Raypa steam autoclave (model AES-75, Barcelona, Spain). Then, the vessel was cooled and opened. The solid and liquid phases were initially separated by gravity filtration using a food-grade fabric with a 300 µm mesh opening (Sefar, Heiden, Switzerland), followed by centrifugation for 30 min at 3750 rpm (Orto Alresa, Madrid, Spain). The crude liquid phase was subsequently concentrated in a stirred ultrafiltration cell operated at 3.5 bar, until a volume concentration factor of 6.25 was achieved. The soluble ulvan fraction was recovered in the retentate of the 10 kDa ultrafiltration membranes (Merk-Millipore, Darmstadt, Germany). The retentate was freeze-dried, milled, and further analyzed before being used as a bioactive ingredient in the formulation of hydrating creams. The detailed workflow is described in Figure 4.

#### 3.2.2. Formulation of a Cream with Green Ingredients

First, a set of preliminary cream formulations was prepared to evaluate their physical appearance, stability, and rheological properties to assess the influence of different thermal spring waters. Moisturizing creams were prepared as oil-in-water (O/W) emulsions with green ingredients according to a standardized formulation protocol [44]. Four formulations were developed: a control cream prepared with distilled water and three experimental creams formulated using thermal waters from the province of Ourense designated as B, L and O. The aqueous phase consisted of distilled water (C) or the corresponding thermal water (73%, *w*/*w*), propanediol (6.0%, *w*/*w*), and Olivem 1000 (Gran Velada, Zaragoza, Spain) (5.0%, *w*/*w*) as the emulsifying agent. Concentrated ulvan was added once dissolved in the aqueous phase. Note here that the ulvan content was selected based on previous optimization studies, as lower concentrations produced overly fluid formulations, whereas concentrations of approximately 2% and 3% caused aggregation and an excessively gel-like consistency, respectively [19]. The oil phase was composed of borage oil (2.0%, *w*/*w*), Rosa mosqueta oil (2.0%, *w*/*w*), shea butter (5.0%, *w*/*w*), and cocoa butter (2.0%, *w*/*w*). The aqueous and oil phases were heated separately to 75 °C under continuous magnetic stirring until complete solubilization and homogenization were achieved. The oil phase was then gradually added to the aqueous phase under continuous stirring to obtain a stable emulsion. The mixture was homogenized for several minutes to ensure uniform dispersion and improve emulsion stability. After emulsification, the formulations were cooled to below 40 °C prior to the addition of the cool-down phase components, including Euxyl PE 9010 (Norderstedt, Germany) preservative (1.0%, *w*/*w*) and hyaluronic acid solution (4.0%, *w*/*w*) (Table 10). The creams were then gently stirred until complete homogenization and stored at room temperature for subsequent rheological analysis.

#### 3.2.3. Formulation and Bottling of Commercial Hydrating Cosmetic Cream

Based on the results obtained, As Burgas thermal spring water was used in the formulation of a commercially prepared cream, with the components and content shown in Table 9. Four types of hand cream were prepared depending on the incorporation of concentrated ulvan and thermal spring water. A placebo cream (DW) was prepared with distilled water, a cream with the same composition, and by replacing distilled water with thermal spring water from As Burgas named TW. To evaluate the impact of ulvan, this ingredient was added at a content optimized in preliminary formulation assays. In samples U-DW and U-TW the content of other functional ingredients (cetearyl alcohol, paraffinum liquidum and isopropyl palmitate) was slightly reduced due to the contribution of ulvan to the viscosity and emollient properties of the creams. All of the creams were bottled following commercial protocols in a cosmetic company. The study with volunteers was performed within the first four months after packing, and the rheological and color measurements were determined after 12 months.

### 3.3. Analytical Methodology

#### 3.3.1. Ulvan

##### Chemical Characterization

Chemical composition of the concentrated ulvan (*n* = 3) was quantified as follows. Some analyses were conducted in the liquid fractions (retentate) prior to freeze-drying, and others on the final dried ulvan extract. The pH was determined under consistent stirring and at room temperature using a Crison PH-25 instrument (CRISON INSTRUMENTS, Barcelona, Spain). The density (g/cm^3^) of the fractions was determined using an Amarell precision hydrometer (Amarell, Municipality, Germany). Total soluble matter in each fraction was calculated using a gravimetric method, after placing samples in an oven at 105 °C until all the aqueous phases evaporated. Ethanol-precipitable matter was isolated from the liquid phases by precipitation with absolute ethanol at a ratio of 1:2, followed by 48 h of storage at 4 °C. Afterwards, mixtures were centrifuged for 15 min at 10.000× *g* and the precipitate was dried at 50 °C until constant weight. Estimated ulvan content was calculated as the ratio of dried ethanol–precipitated matter relative to the weight of retentate used for precipitation.

Ash content was determined by dry extract calcination, according to the standard AOAC Official Method 923.03. Combustion took place at 550 °C for 10 h in a muffle furnace. Both ash and moisture contents (105 °C, 48 h) were gravimetrically quantified.

Protein content was calculated by measuring nitrogen using the Kjeldahl method, with a nitrogen-to-protein conversion factor of 5.13 [45].

The oligosaccharide content in the ulvan was determined by HPLC after a post-hydrolysis stage with 4% sulfuric acid at 121 °C for 20 min and filtration through 0.45 µm membranes. Analyses were performed by HPLC in a 1100 series Agilent chromatograph with a refractive index detector, and Aminex HPX87H column (300 × 7.8 mm, BioRad, Hercules, CA, USA) operating with 0.003 M H_2_SO_4_ as mobile phase (0.6 mL/min) at 60 °C.

The mineral composition was measured after digesting the ashes with hydrogen peroxide and nitric acid (MARSXpress, 1600 W, CEM Corporation, Matthews, NC, USA, 200 °C, 10 min). Macro and trace minerals were quantified by atomic absorption spectroscopy (SpectrAA-220 Fast Sequential, Varian, Mulgrave, Australia), with the exception of K and Na, which were measured by atomic emission, and Cd, which was determined by inductively coupled plasma mass spectrometry (X Series, Thermo Scientific, Waltham, MA, USA).

Sulfate content was measured by ionic chromatography in an Metrohm Advanced IC-861 (Metrohm, Herisau, Switzerland) equipped with a Metrosep A Supp 5–250 column (250 × 4 mm) and IC-819 detector using 3.2 mM sodium carbonate/1 mM sodium bicarbonate (0.70 mL/min) as mobile phase [46].

Total lipid content was quantified according to the Bligh and Dyer method. Briefly, samples were extracted with a chloroform–methanol–water mixture (2:2:1.8, *v*/*v*/*v*), and the lower organic phase was collected, evaporated to dryness, and gravimetrically quantified [47].

Total phenolic content (TPC) was determined following a modified Folin–Ciocalteu method [48], using gallic acid as the standard. Each 500 µL of sample was mixed with 3 mL of distilled water, 500 µL of Folin–Ciocalteu reagent (1 N), and 1 mL of Na_2_CO_3_ (10% *w*/*v*). After 1 h incubation in darkness at room temperature, absorbance was read at 765 nm; the calibration curve was prepared with gallic acid as standard.

##### Structural Analysis

Fourier transform infrared attenuated total reflectance (FTIR-ATR) spectra of the purified ulvan extract were acquired using a NicoletTM 6700 infrared spectrophotometer (Thermo Scientific, MA, USA) equipped with a DTGS detector and a KBr beamsplitter. Spectra were monitored in the range of 4000–400 cm^−1^, averaging 32 scans per sample, using OMNIC software, vs. 7.0 for acquisition and processing.

Molar mass distribution of ulvan extract was measured by High-Performance Size Exclusion Chromatography (HPSEC), equipped with a SuperMultipore PW-H column (6 mm × 15 cm) and a SuperMP (PW)-H guard column (4.6 mm × 3.5 cm) (TSKgel, Tosoh Corporation, Tokyo, Japan). Measurements were made at 40 °C with Milli-Q water (0.4 mL/min). The standard was polyethylene oxide with molecular weights ranging between 12 and 786 kDa (Tosoh Corporation, Japan).

##### Rheological Features

Aqueous solutions of ulvans were prepared following a modified procedure of that previously reported by [10]. Steady state shear viscosity tests of the ulvan solutions were conducted at 25 °C on an MCR302 stress-controlled rheometer (Anton Paar, Graz, Austria) with a sand-blasted plate–plate geometry of 25 mm diameter (0.5 mm gap). The up-and-down curves in terms of the apparent viscosity vs. shear rate were monitored to assess the possible hysteresis effects.

##### Antioxidant Properties

The Trolox Equivalent Antioxidant Capacity (TEAC) was determined [49]. In total, 2 mL of the ABTS^+^ radical solution was mixed with 20 µL of sample or Trolox standard solutions. After incubation for 6 min at 30 °C in the dark, absorbance was read at 734 nm.

#### 3.3.2. Water Sample Preparation and Characterization

The concentrations of metal and metalloid elements were determined by inductively coupled plasma mass spectrometry (ICP-MS). The water samples were filtered with syringe filters (0.45 μm) and subsequently acidified in 1% HNO_3_. Samples were kept in the refrigerator and analyzed with adequate dilution as needed.

#### 3.3.3. Characterization of Hand Creams

##### Physicochemical Analysis: Physical Appearance, pH and Viscosity

Color measurements were performed at room temperature using a Tristimulus colorimeter (Minolta CR-400, Konica Minolta, Tokyo, Japan). The CIELab color space parameters were determined, the lightness L* varying from 0, whiteness to 100, brightness, a* from <0, greenness to >0, redness, and b* from >0, blueness to >0, yellowness. The color differences (ΔE) were calculated according to Equation (1). All hand creams were subjected to a tenfold evaluation at the same light conditions:

(1) $$\Delta E^{*}=\sqrt{{\left({\Delta L}^{*}\right)}^{2}+{\left({\Delta a}^{*}\right)}^{2}+{\left({\Delta b}^{*}\right)}^{2}}$$

pH was measured using a pH meter (Crison, Alella, Spain).

The apparent viscosity *vs.* shear rate of the formulated creams was monitored by means of steady state shear measurements. The tests were conducted on an MCR302 controlled-stress rheometer using the aforementioned geometry (1 mm gap) at 25 °C. All creams were rested between the plates for 5 min to allow for thermal and structural homogenization of the samples. Note here that potential hysteresis effects were also analyzed. All of the above measurements were made at least in duplicate.

#### 3.3.4. Efficacy Assessment of the Hand Creams

##### Study Design

This is a quasi-experimental, pre–post pilot study conducted in a university setting with healthy volunteers affiliated with the Nursing Degree program (students, faculty, and administrative and support staff). The study flow diagram is presented in Figure 3.

##### Study and Recruitment Period

The study period spanned from November 2024 to March 2025. Recruitment was conducted via email invitations through authorized mailing lists, as well as through informational meetings aimed at the university community. Data collection and initial measurements were carried out during February and March 2025.

##### Inclusion and Exclusion Criteria

Women and men aged 18–65, affiliated with the university’s Nursing degree program, who were able to consent to their participation, had no active pathologies, and whose hands were free of lesions or wounds, were included. Subjects were excluded if they presented any of the following criteria: allergies or hypersensitivity to topical skin products, simultaneous participation in another clinical trial with cosmetics or in other experimental studies, or failure to sign the informed consent form.

##### Products and Group Allocation

The four products shared the same base formulation (unscented hand cream), differing only in the added active ingredients. Group assignment was based on the order of recruitment:

Group 1—Control (DW): Base hand cream prepared with distilled water, without any additional active ingredient.

Group 2—Thermal (TW): Base hand cream prepared with 70.6% of thermal spring water.

Group 3—Ulvan (U-DW): Base hand cream prepared with distilled water, with 1% refined ulvan extract from *Ulva* spp.

Group 4—Ulvan+Thermal (U-TW) (main experimental group): Base hand cream with 72.0% thermal spring water and 1% ulvan extract from *Ulva* spp.

Each participant received a pump dispenser according to their assigned group.

##### Intervention

During the initial appointment, the study’s objective and procedure were explained, informed consent was obtained, the first dermatological measurements were taken using a skin analyzer, and the relevant questionnaires were completed. Each participant used the cosmetic sample provided every night for 14 consecutive days, applying it to both hands before going to bed. At the end of the intervention period, all initial measurements were repeated to evaluate the effects of the intervention. The 14-day period was selected based on evidence from previous studies on mineral–medicinal water applications [50,51].

##### Variables

The following variables were collected:

**Sociodemographic and epidemiological data:** Age, gender, and occupation.

**Smoking status:** Smoker/nonsmoker.

**Hygiene and skin care habits:** Frequency of handwashing (<5, 5–10, or >10 times per day), use of hand cream (never, occasionally, or always), and use of gloves.

**Objective variable:** Skin hydration was measured using the Corneometer^®^ CM 825 (Courage+Khazaka Electronic GmbH, Cologne, Germany), a non-invasive capacitive measurement device. Three consecutive measurements were taken on the back of the dominant hand, and the mean value was recorded. The results are expressed in Arbitrary Units (AU) on a scale of 0 to 120 [52]. To allow for direct comparison with subjective perception, the values were further classified into three categories: very dry (<30 AU), dry (30–45 AU), and normal/hydrated (>45 AU). These category ranges were adapted according to the standard ranges previously described by Constatin et al. [34].

**Subjective variables:** Perception of skin hydration status (five-category ordinal scale: very dry, dry, normal, hydrated, and very hydrated; for comparison with the objective measurement, this was grouped into three equivalent categories: very dry, dry, and normal/hydrated), sensation of tightness (none, mild, moderate, and severe), perception of scaling (no/yes), and perception of irritation or redness (no/yes).

##### Calculation of the Sample Size

The sample size was determined using EPIDAT 4.0 software. Expecting positive differences in hydration levels between the Ulvan+Thermal group and the control group, it was estimated that a minimum of 22 participants per group would be needed to detect a significant difference, with a 95% confidence level and 80% power. The 95% confidence intervals for median changes (delta PRE → POST) were calculated using bootstrap resampling with 10,000 iterations. The 95% confidence intervals for Rosenthal’s r were obtained using Fisher’s z-transformation. Considering possible losses during follow-up (maximum 20%), 30 subjects were included per group, for an initial total of 120 participants. Given the pilot nature of the study and the absence of prior data on this specific formulation, the calculation was based on the estimation of a minimum clinically relevant difference in objective skin hydration.

To formally assess the interaction between ulvan and thermal water on skin hydration, the Scheirer–Ray–Hare test was applied to the objective hydration delta (PRE→POST). This non-parametric equivalent of a two-way ANOVA evaluates the main effects of ulvan (yes/no) and thermal water (yes/no), as well as their interaction term (Ulvan × Thermal Water), on the ranked delta values. The test statistic H was calculated as the ratio of the sum of squares for each factor to the total sum of squares divided by the total sample size, and its significance was evaluated using the chi-squared distribution.

##### Ethical Considerations

The study received a favorable opinion from the Research Ethics Committee of A Coruña-Ferrol (Registration Code 2024/169) and was conducted in accordance with the principles of the Declaration of Helsinki. All participants were informed about the objectives and procedures of the study and signed an informed consent form before their inclusion.

### 3.4. Statistical Analysis

Experimental data were evaluated using a one-factor analysis of variance; a Scheffé test was conducted using v.22 PASW Statistics software (IBM SPSS Statistics, IBM, New York, NY, USA) if differences between mean values were found.

For the volunteer study, statistical analysis was performed using IBM SPSS Statistics version 22.0. The normality of the continuous hydration variable (AU) was verified using the Shapiro–Wilk test, which revealed non-normal distributions in most groups and measurement times. Given the ordinal nature of the main variables and the lack of normality in the continuous variable, non-parametric tests were used in all analyses. The main statistical analysis was performed on the continuous AU values. Baseline homogeneity between groups was verified using the Kruskal–Wallis test for ordinal and continuous variables and the Pearson chi-square test for categorical variables. Within-group change (PRE → POST) was analyzed using the Wilcoxon signed-rank test. The post-intervention intergroup comparison was performed using the Kruskal–Wallis test, followed by post hoc comparisons using the Mann–Whitney U test. In both cases, the Bonferroni correction for multiple comparisons was applied: adjusted α = 0.0125 for within-group comparisons (4 groups) and adjusted α = 0.0083 for between-group post hoc comparisons (6 pairs). For the continuous variable of hydration, individual change was further analyzed using the pre–post change (Δ = post − pre). For the dichotomous tolerability variables (scaling and irritation), within-group change was assessed using the exact McNemar test, and between-group comparisons were assessed using Pearson’s chi-squared test, with Cramer’s V as the effect size measure. Effect size was quantified using Rosenthal’s r for the Wilcoxon and Mann–Whitney U tests [53], and using η^2^ for the Kruskal–Wallis test [54]. Reference values for interpreting r were: small effect > 0.1, medium effect > 0.3, and large effect > 0.5. The level of statistical significance was set at *p* < 0.05. To formally assess the interaction between the ulvan and thermal water factors on skin hydration, the Scheirer–Ray–Hare test was additionally applied to the objective hydration delta (PRE→POST), as a non-parametric equivalent of a two-way ANOVA. This test evaluates the main effects and the interaction term (Ulvan × Thermal Water) on ranked values, with the test statistic H evaluated against the chi-squared distribution.

## 4. Conclusions

A concentrated ulvan product was obtained with an aqueous chemical-free extraction and purification process. The incorporation of ulvan was tested in model cosmetic creams with green ingredients and different local thermal spring waters that were low in mineralization, rich in bicarbonate sodium, and also contained sulfate. The rheological results suggest that the incorporation of thermal spring water may facilitate application to the skin and improve spreadability. The individual effects of ulvan and As Burgas thermal spring water, as well as their additive effect—confirmed by the Scheirer–Ray–Hare test to be additive rather than synergistic—were evaluated on healthy volunteers. The skin hydration in the Ulvan+Thermal group was significantly improved over the control with distilled water and no ulvan, and both objective and subjective evaluation were coincident. A high tolerance was observed, since all of the tested creams reduced the tightness sensation, whereas the scaling was significantly reduced in the ulvan group. The results provide preliminary evidence on the potential skin benefits of combining ulvan with thermal spring water in hand cream formulations. These are very promising results, and in a future study, it is be recommended to evaluate the effects on volunteers with skin affectation, pathologies, or with exposure to handwashing and glove use, as well as to further explore the potential of these renewable ingredients in therapeutic skin applications.

This study has several limitations that should be considered when interpreting the results. First, the quasi-experimental design did not include the blinding of investigators or outcome assessors. However, all formulations were presented to participants in identical dispensers differentiated only by an identification code known exclusively by the investigators, ensuring complete blinding of participants with respect to the assigned product, which minimized the risk of expectancy bias in subjective assessments. Second, the 14-day intervention period, while sufficient to detect changes in surface hydration, does not allow for the evaluation of medium- or long-term effects or the persistence of the observed changes after product withdrawal. Third, the Corneometer^®^ measurements were not performed under standardized ambient temperature and humidity conditions, which could introduce some variability in the absolute values, although the random assignment of groups minimizes the impact of this on the comparisons. Fourth, the sample size, while sufficient to detect differences in the primary variable, limited the statistical power to detect significant changes in variables with a low frequency of occurrence, such as scaling and irritation. Finally, the sample, composed mainly of young female nursing students with frequent exposure to handwashing and glove use, limits the generalizability of the results to other populations. Future studies with larger sample sizes, longer follow-up periods, and more heterogeneous populations would allow for the confirmation and expansion of the findings of this study. Additionally, the Scheirer–Ray–Hare test confirmed that the observed effect is additive rather than synergistic; future studies employing factorial designs with formal interaction analyses would allow for a more rigorous evaluation of potential synergistic effects. Furthermore, the absence of additional objective measures such as transepidermal water loss (TEWL), skin elasticity, or lipid barrier biomarkers limits the comprehensiveness of the efficacy evaluation. Finally, the proposed mechanisms of action remain speculative, as no experimental data on skin penetration or molecular pathways were collected; these represent important directions for future research.

## Figures and Tables

**Table 1:** Composition of the concentrated ulvan extract.

Component	Content (g/100 g Ulvan) ^1^	Element	Content (ppm) ^2^
**pH**	6.03 ± 0.11	**Macro and micro minerals**	
**Moisture**	10.88 ± 2.18	Calcium (Ca)	7287.02
**Carbohydrates**	63.4 ± 5.0	Potassium (K)	16,692.16
Rhamnose	30.9 ± 3.1	Magnesium (Mg)	40,543.01
Glucose	14.3 ± 0.4	Sodium (Na)	6857.49
Xylose	7.5 ± 0.5	Iron (Fe)	86.24
Glucuronic acid	4.2 ± 0.2	Manganese (Mn)	20.34
Iduronic acid	6.5 ± 0.8	Copper (Cu)	0.45 *
**Protein**	6.82 ± 0.43	Zinc (Zn)	1.19
**Lipids**	0.91 ± 0.13	Cobalt (Co)	0.01 *
**Ash**	19.81 ± 0.41	Nickel (Ni)	1.14
Sulphate	9.65 ± 0.93	**Heavy metals**	
**Total phenolics** (**mg GAE/g DW**)	2.70 ±0.27	Arsenic (As)	0.72 *
		Cadmium (Cd)	0.02 *
ABTS^+^ RSA ^3^ (% inhibition)	75.49 ± 4.17	Lead (Pb)	0.22 *
Ethanol-precipitable matter	97.90 ± 2.05	Mercury (Hg)	0.00 *

^1^ Wet basis. ^2^ Coefficient of variation < 2.5%. ^3^ Radical scavenging activity. * Below detection limit.

**Table 2:** Characteristics of color, pH, rheology, and microbiological quality of commercial hydrating creams. Color results represent average ± standard deviation (*n* = 10). Time is expressed in months.

	Control (DW)		Thermal (TW)		Ulvan (U-DW)		Ulvan+Thermal (U-TW)	
**Color**	t = 0	t = 12	t = 0	t = 12	t = 0	t = 12	t = 0	t = 12
L*	94.2 ± 0.5	92.8 ± 0.4	93.5 ± 1.2	92.9 ± 1.3	93.1 ± 1.5	92.2 ± 1.0	92.2 ± 1.4	91.6 ± 1.2
a*	−1.9 ± 0.1	−2.2 ± 0.2	−1.7 ± 0.3	−1.5 ± 0.1	−1.8 ± 0.1	−1.7 ± 0.3	−1.6 ± 0.3	−1.9 ± 0.2
b*	4.4 ± 0.2	3.9 ± 0.3	3.8 ± 0.3	3.6 ± 0.2	4.1 ± 0.2	3.9 ± 0.3	3.9 ± 0.2	3.6 ± 0.1
**pH**	5.5	5.5	6.0	6.1	6.0	6.1	6.0	6.2
**Rheology** (**mPa·s**) *	[2.26–2.13] 10^5^		[5.52–5.35] 10^5^		[3.98–3.90] 10^5^		[3.32–3.28] 10^5^	
**Microbiological quality** (**Total aerobic microbial count**)								
*Escherichia coli*	Absence							
*Pseudomonas aeruginosa*	Absence							
*Staphylococcus aureus*	Absence							
*Candida albicans*	Absence							

* Viscosity at 0.1 s^−1^ corresponds to the value at time = 0 and after 1 year of storage at RT.

**Table 3:** Baseline characteristics of the sample by group.

Variable	Control (*n* = 29) n (%) or Mean Value ± SD	Thermal (*n* = 29) n (%) or Mean Value ± SD	Ulvan (*n* = 30) n (%) or Mean Value ± SD	Ulvan+Thermal (*n* = 30) n (%) or Mean Value ± SD
**Age** (**years**)				
Mean ± SD	27.3 ± 14.3	26.2 ± 11.3	25.7 ± 12.0	26.8 ± 13.2
Median (range)	21 (18–63)	22 (18–56)	21 (18–63)	21 (18–61)
Kruskal–Wallis	H = 0.157		*p* = 0.984	
**Gender**				
Female	3 (10.3)	2 (6.9)	2 (6.7)	4 (13.3)
Male	26 (89.7)	27 (93.1)	28 (93.3)	26 (86.7)
Chi-square	χ^2^ = 1.059		*p* = 0.787	
**Occupation**				
Student	25 (86.2)	24 (82.8)	26 (86.7)	26 (86.7)
Teaching	1 (3.4)	2 (6.9)	2 (6.7)	1 (3.3)
Administration	3 (10.3)	3 (10.3)	2 (6.7)	3 (10.0)
Chi-square	χ^2^ = 1.012		*p* = 0.985	
**Hand care habits**				
Washing frequency: No	0 (0.0)	1 (3.4)	0 (0.0)	0 (0.0)
Washing frequency: <5/day	4 (13.8)	1 (3.4)	4 (13.3)	1 (3.3)
Washing frequency: 5–10/day	14 (48.3)	15 (51.7)	14 (46.7)	13 (43.3)
Washing frequency: >10/day	11 (37.9)	12 (41.4)	12 (40.0)	16 (53.3)
Chi-square	χ^2^ = 7.892		*p* = 0.545	
Use of cream: Never	8 (27.6)	10 (34.5)	11 (36.7)	8 (26.7)
Use of cream: Occasional	17 (58.6)	12 (41.4)	18 (60.0)	12 (40.0)
Use of cream: Always	4 (13.8)	7 (24.1)	1 (3.3)	10 (33.3)
Chi-square	χ^2^ = 10.844		*p* = 0.093	
Use of gloves: Yes	19 (65.5)	19 (65.5)	19 (63.3)	20 (66.7)
Chi-square	χ^2^ = 0.077		*p* = 0.994	
**Smoking habit**				
Smoker: Yes	2 (6.9)	4 (13.8)	3 (10.0)	2 (6.7)
Smoker: No	27 (93.1)	25 (86.2)	27 (90.0)	28 (93.3)

**Table 4:** Objective skin hydration by group prior to (PRE) and after (POST) application of creams.

	Control (*n* = 29)	Thermal (*n* = 29)	Ulvan (*n* = 30)	Ulvan+Thermal (*n* = 30)
PRE (AU) ^1^	39.0 (34.0–43.0)	41.0 (32.0–44.0)	38.0 (35.0–42.3)	38.0 (36.3–39.0)
POST (AU) ^1^	39.0 (33.0–43.0)	46.0 (34.0–47.0)	46.0 (45.0–48.8)	67.5 (62.0–69.8)
Δ median (AU) ^2^	0.0	4.0	8.5	28.0
IC 95% Δ	[0.0; 0.0]	[3.0; 5.0]	[6.5; 11.0]	**[24.0; 29.5]**
**Statistics**				
Basal homogeneity—Kruskal–Wallis PRE (4 groups)				
Global PRE		H = 0.809	*p* = 0.847	η^2^ = 0.007 ^5^
Intragroup comparisons PRE→POST—Wilcoxon (α Bonferroni = 0.0125)				
U	-	-	-	-
Z	−1.265	−4.718	−4.705	−4.766
*p*	0.206	<0.001	<0.001	<0.001
Effect size ^3^	-	r = 0.876	r = 0.859	r = 0.870
IC 95% ^4^	[−0.144; 0.554]	[0.751; 0.941]	[0.722; 0.931]	[0.743; 0.937]

^1^ Values are presented as median (IQR), where IQR is the interquartile range. ^2^ Δ median = POST − PRE objective skin hydration increment expressed in Arbitrary Units (AU). ^3^ Effect size magnitude: small r ≥ 0.10; medium r ≥ 0.30; large r ≥ 0.50. ^4^ IC 95% for the delta via bootstrapping (10,000 resamples) using Fisher’s z transformation. ^5^ η^2^ = H/(N − 1).

**Table 5:** Change in objective hydration (∆ PRE → POST) by group.

	Control (*n* = 29)		Thermal (*n* = 29)		Ulvan (*n* = 30)	Ulvan+Thermal (*n* = 30)
Δ median (AU) ^1^	0.0		4.0		8.5	28.0
Interquartile range (IQR)	0.0–0.0		3.0–7.0		4.3–11.8	23.3–30.8
IC 95% Δ	[0.0; 0.0]		[3.0; 5.0]		[6.5; 11.0]	[24.0; 29.5]
	**Statistics**					
	Global comparative—Kruskal–Wallis delta (4 groups)					
Global (Δ median)		H = 90.248	*p* < 0.001			η^2^ = 0.771 ^4^
	Comparisons post hoc—Mann–Whitney U (α Bonferroni = 0.0083)					
	U	Z	*P*	*p* _adjusted_	Rosenthal’s r effect size ^2^	IC 95% r ^3^
Control *vs.* Thermal	3.5	−6.685	<0.001	0.001	r = 0.878	[0.801; 0.926]
Control *vs.* Ulvan	20.0	−6.491	<0.001	0.001	r = 0.845	[0.752; 0.905]
Control *vs.* Ulvan+Thermal	28.0	−6.342	<0.001	0.030	r = 0.826	[0.722; 0.893]
Thermal *vs.* Ulvan	251.0	−2.801	0.005	0.030	r = 0.365	[0.120; 0.568]
Thermal *vs.* Ulvan+Thermal	29.0	−6.169	<0.001	0.001	r = 0.803	[0.689; 0.879]
Ulvan *vs.* Ulvan+Thermal	45.0	−5.993	<0.001	0.001	r = 0.774	[0.647; 0.859]

^1^ Δ median = POST − PRE objective skin hydration increment expressed in Arbitrary Units (AU). ^2^ Effect size magnitude: small r ≥ 0.10; medium r ≥ 0.30; large r ≥ 0.50. ^3^ IC 95% for the delta via bootstrapping (10,000 resamples) using Fisher’s z transformation. ^4^ η^2^ = H/(N − 1).

**Table 6:** Comparison between measured objective hydration (Obj.) and subjective perception of skin hydration (Sub.) by group. The column n indicates number of subjects in each category, whereas % indicates the percentage of subjects relative to total number of participants in each group (*n* = 29; 29; 30 and 30).

	Control (*n* = 29)				Thermal (*n* = 29)				Ulvan (*n* = 30)				Ulvan+Thermal (*n* = 30)			
	Obj.		Sub.		Obj.		Sub.		Obj.		Sub.		Obj.		Sub.	
**Category ^1^**	n	%	n	%	n	%	n	%	n	%	n	%	n	%	n	%
**PRE**																
Very dry	6	20.7	2	6.9	7	24.1	2	6.9	5	16.7	1	3.3	4	13.3	1	3.3
Dry	21	72.4	8	27.6	21	72.4	4	13.8	19	63.3	2	6.7	25	83.3	8	26.7
Normal/hydrated	2	6.9	19	65.5	1	3.4	23	79.3	6	20.0	27	90.0	1	3.3	21	70.0
**POST**																
Very dry	5	17.2	1	3.4	0	0.0	0	0.0	1	3.3	0	0.0	0	0.0	1	3.3
Dry	21	72.4	11	37.9	10	34.5	3	10.3	8	26.7	0	0.0	1	3.3	0	0.0
Normal/hydrated	3	10.3	17	58.6	19	65.5	26	89.7	21	70.0	30	100	29	96.7	29	96.7

^1^ Hydration categories: very dry AU < 30; dry AU ϵ [30;45]; normal/hydrated AU > 45.

**Table 7:** Subjective perception of skin hydration by group. The column n indicates number of subjects in each category, whereas % indicates the percentage of subjects relative to total number of participants in each group (*n* = 29; 29; 30 and 30).

	Control (*n* = 29)				Thermal (*n* = 29)				Ulvan (*n* = 30)				Ulvan+Thermal (*n* = 30)			
	PRE		POST		PRE		POST		PRE		POST		PRE		POST	
**Category**	n	%	n	%	n	%	n	%	n	%	n	%	n	%	n	%
Very dry	2	6.9	1	3.4	2	6.9	0	0.0	1	3.3	0	0.0	1	3.3	1	3.3
Dry	8	27.6	11	37.9	4	13.8	3	10.3	2	6.7	0	0.0	8	26.7	0	0.0
Normal	12	41.1	8	27.6	17	58.6	2	6.9	22	73.3	6	20.0	16	53.3	6	20.0
Hydrated	7	24.1	8	27.6	6	20.7	22	75.9	5	16.7	18	60.0	5	16.7	21	70.0
Very hydrated	0	0.0	1	3.4	0	0.0	2	6.9	0	0.0	6	20.0	0	0.0	2	6.7
**Statistics**																
Basal homogeneity—Kruskal–Wallis PRE (4 groups)																
Global PRE					H = 1.641				*p* = 0.564				η^2^ = 0.014			
Intragroup variation PRE→POST—Wilcoxon (α Bonferroni = 0.0125)																
	U				Z				*p*		*p* _adjusted_		Rosenthal’s r effect size			
Control	—				−0.533				0.564		1		—			
Thermal	—				−3.397				<0.001		0.004		r = 0.631			
Ulvan	—				−4.197				<0.001		<0.001		r = 0.766			
Ulvan+Thermal	—				−3.771				<0.001		<0.001		r = 0.689			
Intergroup comparison POST—Kruskal–Wallis + Mann–Whitney U (α Bonferroni = 0.0083)																
Global POST					H = 25.420				*p* < 0.001				η^2^ = 0.217			
	U				Z				*p*		*p* _adjusted_		Rosenthal’s r effect size			
Control *vs.* Thermal	204.5				−3.359				<0.001		0.006		r = 0.441			
Control *vs.* Ulvan	171.0				−4.003				<0.001		<0.001		r = 0.521			
Control *vs.* Ulvan+Thermal	212.5				−3.374				<0.001		0.006		r = 0.439			
Thermal *vs.* Ulvan	390.0				−0.682				0.414		1		—			
Thermal *vs.* Ulvan+Thermal	454.0				0.288				0.720		1		—			
Ulvan *vs.* Ulvan+Thermal	513.0				0.931				0.275		1		—			

**Table 8:** Skin tightness: Distribution and statistics by group. The column n indicates number of subjects in each category, whereas % indicates the percentage of subjects relative to total number of participants in each group (*n* = 29; 29; 30 and 30).

	Control (*n* = 29)				Thermal (*n* = 29)				Ulvan (*n* = 30)				Ulvan+Thermal (*n* = 30)			
	PRE		POST		PRE		POST		PRE		POST		PRE		POST	
**Category**	n	%	n	%	n	%	n	%	n	%	n	%	n	%	n	%
None	17	58.6	25	86.2	13	44.8	23	79.3	15	50.0	25	83.3	14	46.7	22	73.3
Mild	8	27.6	2	6.9	12	41.4	5	17.2	13	43.3	4	13.3	10	33.3	5	16.7
Moderate	3	10.3	2	2.9	4	13.8	1	3.4	2	6.7	1	3.3	6	20.0	3	10.0
Severe	1	3.4	0	0.0	0	0.0	0	0.0	0	0.0	0	0.0	0	0.0	0	0.0
**Statistics**																
Basal homogeneity—Kruskal–Wallis PRE (4 groups)																
Global PRE					H = 1.233				*p* = 0.745				η^2^ = 0.009			
Intragroup variation PRE→POST—Wilcoxon (α Bonferroni = 0.0125)																
					Z				*p*		*p*_adjusted_		Rosenthal’s r effect size			
Control					−2.484				0.013		0.052		r = 0.461			
Thermal					−2.476				0.013		0.052		r = 0.460			
Ulvan					−2.517				0.012		0.048		r = 0.460			
Ulvan+Thermal					−2.840				0.005		0.020		r = 0.519			
Intergroup comparison POST—Kruskal–Wallis (α Bonferroni = 0.0083)																
Global POST					H = 1.748				*p* = 0.626				η^2^ = 0.015			

**Table 9:** Skin scaling and irritation by group. The column n indicates number of subjects in each category, whereas % indicates the percentage of subjects relative to total number of participants in each group (*n* = 29; 29; 30 and 30).

	Control (*n* = 29)				Thermal (*n* = 29)				Ulvan (*n* = 30)				Ulvan+Thermal (*n* = 30)			
	PRE		POST		PRE		POST		PRE		POST		PRE		POST	
**Variable**	n	%	n	%	n	%	n	%	n	%	n	%	n	%	n	%
**Scaling**																
Yes	8	27.6	7	24.1	7	24.1	2	6.9	9	30.0	3	10.0	7	23.3	2	6.7
**Statistics**																
McNemar (PRE → POST)	*p* = 1				*p* = 0.062				*p* = 0.031				*p* = 0.125			
Chi^2^ POST among groups					χ^2^ = 5.737				V = 0.220				*p* = 0.125			
**Irritation**																
Yes	5	17.2	1	3.4	6	20.7	1	3.4	4	13.3	2	6.7	4	13.3	0	0.0
**Statistics**																
McNemar (PRE → POST)	*p* = 0.125				*p* = 0.062				*p* = 0.625				*p* = 0.125			
Chi^2^ POST among groups					χ^2^ = 2.037				V = 0.131				*p* = 0.565			

**Table 10:** Composition of hydrating cosmetic creams formulated with green ingredients.

Hydrating Cream with Green Ingredients			Commercial Hydrating Cream		
Component		Content (%, *w*/*w*)	Component	Content (%, *w*/*w*) DW, TW	Content (%, *w*/*w*) U-DW, U-TW
**Aqueous phase**			Water	70.59	72.04
	Water (DW; B; L; O)	73.00	Cetearyl alcohol	8.00	7.14
	Propanediol	6.00	Paraffinum liquidum	5.00	4.08
	Olivem 1000	5.00	Isopropyl palmitate	5.00	4.08
	Ulvan	1.00	Steareth-21	5.00	5.10
**Oil phase**			Glycerin	4.97	5.07
	Shea butter	5.00	Phenoxyethanol	0.92	0.94
	Borage oil	2.00	Hydroxypropyl starch phosphate	0.20	0.20
	Rosa mosqueta oil	2.00	Caprylyl glycol	0.12	0.12
	Cocoa butter	2.00	Disodium EDTA	0.10	0.10
	Euxyl PE 9010	1.00	Potassium cetyl phosphate	0.09	0.09
	Hyaluronic acid	4.00	Ulvan	-	1.02

## Data Availability

All data generated or analyzed during this study are included in this published article.

## Abbreviations

The following abbreviations are used in this manuscript:

DW	Placebo cream, with distilled water and no additional active ingredient
TW	Thermal base cream with 70.6% thermal spring water
U-DW	Ulvan base cream with 1% ulvan extract from *Ulva* spp. and distilled water
U-TW	Ulvan (1%) + Thermal base cream with 72% of As Burgas thermal water
(B)	Thermal water from As Burgas
(L)	Thermal water from Laias
(O)	Thermal water from Outariz

## Appendix A

**Table A1:** Mineral composition of the thermal spring waters used in the study.

Component (mg/L)	As Burgas (B)	Laias (L)	Outariz (O)
Na	220.35	200.50	224.56
K	8.477	6.656	8.548
Ca^2+^	8.417	6.121	8.904
Mg^2+^	0.720	0.513	0.761
HCO_3_^−^	462.0	488.0	292.0
Cl^−^	25.0	19.7	19.0
SO_4_^2−^	3.0	26.0	14.0
As	0.009	0.009	0.007
Cu	0.000	0.002	0.000
Fe	0.000	0.001	0.004
Mn	0.028	0.026	0.028
Ni^2+^	0.000	0.002	0.001
Se	0.001	0.004	0.003
Zn	0.001	0.001	0.001
Al	0.003	0.000	0.000
B	0.608	0.979	0.632
Ba	0.004	0.009	0.006
Be	0.001	0.000	0.001
Cs	39.170	30.800	26.520
Li	0.857	0.733	0.859
Rb	0.144	0.122	0.150
Sr	0.076	0.078	0.082
V	0.002	0.001	0.002
